# Inpatient or Outpatient: Does Initial Disposition Affect Outcomes in Trimalleolar Ankle Fractures?

**DOI:** 10.7759/cureus.59586

**Published:** 2024-05-03

**Authors:** Samantha Baxter, Andrea H Johnson, Jane C Brennan, Parimal Rana, Elizabeth Friedmann, Adrienne Spirt, Justin J Turcotte, David Keblish

**Affiliations:** 1 Orthopedic Research, Anne Arundel Medical Center, Annapolis, USA; 2 Orthopedics, Anne Arundel Medical Center, Annapolis, USA; 3 Orthopedic Surgery, Anne Arundel Medical Center, Annapolis, USA; 4 Orthopedic and Surgical Research, Anne Arundel Medical Center, Annapolis, USA

**Keywords:** length of stay, postoperative outcomes, outpatient care, inpatient care, trimalleolar ankle fracture

## Abstract

Background

The repair of trimalleolar fractures can be challenging for surgeons and may be managed as an inpatient or an outpatient. However, it is often unclear whether these patients should be admitted immediately or sent home from the emergency department (ED). This study aims to evaluate trimalleolar fractures treated surgically in the inpatient or outpatient settings to evaluate differences in outcomes for these patients.

Methods

A retrospective chart review of 223 patients undergoing open reduction internal fixation of a trimalleolar ankle fracture was performed from January 2015 to August 2022. Patients were classified by whether the fixation was performed as an inpatient or outpatient. Outcomes of interest included time from injury to surgery, complications, ED returns, and readmissions within 90 days.

Results

Inpatients had significantly higher ASA scores, BMI, and rates of comorbidities. Inpatient treatment was associated with faster time to surgery (median 2.0 vs. 9.0 days) and fewer delayed surgeries more than seven days from injury (18.4 vs. 67.9%). There were no differences in complications, 90-day ED returns, readmissions, or reoperation between groups.

Conclusions

Inpatient admission of patients presenting with trimalleolar ankle fractures resulted in faster time to surgery and fewer surgical delays than outpatient surgery. Despite having more preoperative risk factors, inpatients experienced similar postoperative outcomes as patients discharged home to return for outpatient surgery. Less restrictive admission criteria may improve the patient experience by providing more patients with support and pain control in the hospital setting while decreasing the time to surgery.

## Introduction

Ankle fractures represent a common injury pattern managed by orthopedic surgeons. Their incidence has been estimated at 174 per 100,000 adults annually [1]. A recently published study projected the incidence of trimalleolar fractures, specifically, to be 40 per 100,000 adults each year, with the peak occurring from age 60 to 69 [2,3]. Given the unstable nature of trimalleolar fractures, operative repair is generally indicated; however, the timing for repair has been debated. A case series evaluating timing to fixation noted an increase in infection risk by a factor of six in the delayed group regardless of the length of the delay [4]. In contrast, careful monitoring of soft tissue status may allow delayed operative fixation without complication [5].

Decisions regarding time to operation depend on a variety of factors. Elements such as age, body mass index (BMI), American Society of Anesthesiologists (ASA) class, diabetes, and hypertension (HTN) have been shown to be independently associated with admission from the emergency department (ED) and inpatient repair of ankle fractures [6]. Conversely, if a patient has few medical conditions, surgeons may opt to perform the repair as an outpatient to give time for soft tissue swelling to decrease. Admission and inpatient repair are associated with an increase in healthcare costs compared to outpatient management, which may place a strain on healthcare resources [7,8]. While significant variability in ankle fracture management exists broadly, one study determined that costs can be decreased when ankle fractures are approached using a standardized care pathway [9]. Similarly, a structured approach to outpatient management may preserve healthcare resources with minimal effect on infection risk and negative outcomes for closed ankle fractures [10]. Outpatient management may decrease costs without increasing negative outcomes for patients and may be combined with home care services and day-surgery units to manage patient status leading up to surgery [11,12]. However, the decision to admit or manage as an outpatient is one that should be considered carefully by the surgeon. This study aims to evaluate trimalleolar fractures treated surgically in the inpatient or outpatient settings to evaluate differences in outcomes for these patients.

## Materials and methods

This study was approved by the institutional review board as an exempt review of existing medical records. A retrospective chart review of all patients who had an open reduction and internal fixation (ORIF) of their trimalleolar ankle fracture between January 2015 and August 2022 at a single institution was performed. In total, 223 patients were included in the study.

Independent variables

Independent variables of interest included age, BMI, race, sex, and ASA score. Comorbidities of interest included atrial fibrillation, asthma, anxiety, anemia, coronary artery disease (CAD), congestive heart failure (CHF), coagulation defects, chronic obstructive pulmonary disease (COPD), dementia, depressive disorder, end-stage renal disease (ESRD), gastroesophageal reflux disease (GERD), HTN, liver disease, malnutrition, mania/bipolar disorder, neoplasm, osteoarthritis, obesity/overweight, rheumatoid arthritis, sleep apnea, type one diabetes, type two diabetes, vascular disease, and hierarchical condition category (HCC) score. Injury mechanism type, time from injury to surgery, preoperative delay greater than seven days, the reason for the preoperative delay, functional status, and minutes in the operating room were also evaluated.

Outcome measures

Outcomes of interest included length of stay, discharge home, 90-day post-op ED visit, time to ED visit, 90-day readmission, time to readmission, any reoperation, unplanned reoperation, time to reoperation, reason for reoperation, postoperative complication, complication type, and months to last orthopedic follow-up. A negative outcome is defined as a 90-day ED visit, 90-day readmission, complication, or unplanned reoperation.

Statistical analysis

Patients were classified by whether they had their ORIF inpatient or outpatient. Univariate analysis, including chi-square tests and two-sided independent samples t-tests, were used to determine demographic/comorbidity, injury characteristics, surgery detail, and postoperative outcome differences between groups. Fisher’s exact test was performed when the assumptions of chi-square testing were not met. Mood’s test of medians and one-sided t-tests were used where appropriate. Multivariate logistic regression was used to assess predictors of a negative outcome (version 1.4.1717© 2009-2021 RStudio, PBC). Statistical significance was assessed at p < 0.05.

## Results

Of the 222 patients, 38 (17.1%) underwent ORIF as an inpatient at the time of presentation and 184 (82.9%) underwent outpatient ORIF. On average, inpatients were older (66.29 ± 15.52 vs. 51.32 ± 16.72; p < 0.001) and had a higher BMI (33.74 ± 10.82 vs. 29.49 ± 6.35; p = 0.024) than those who had their surgery in an outpatient setting. Additionally, those who were inpatient had a higher proportion of patients who had an ASA score over 3 (76.3% (29) vs. 17.4% (32); p < 0.001) and higher average HCC scores (1.03 ± 1.11 vs. 0.38 ± 0.12; p = 0.006), indicating greater overall comorbidity burden. With regards to specific comorbidities, patients treated on an inpatient basis had a higher prevalence of atrial fibrillation, anemia, CAD, CHF, COPD, dementia, ESRD, GERD, HTN, sleep apnea, and type 2 diabetes (all p < 0.05) (Table 1).

**Table 1:** Patient demographics and comorbidities Data are expressed as mean ± SD or n (%); p-values < 0.05 in bold BMI, body mass index; ASA, American Society of Anesthesiologists score; CAD, coronary artery disease; CHF, congestive heart failure; COPD, chronic obstructive pulmonary disease; ESRD, end-stage renal disease; GERD, gastroesophageal reflux disease; HTN, hypertension; HCC, hierarchical condition category

Demographics and comorbidities	Inpatient (n = 38)	Outpatient (n = 184)	p-value
Age	66.29 ± 15.52	51.32 ± 16.72	<0.001
BMI	33.74 ± 10.82	29.49 ± 6.35	0.024
Non-White	6 (15.8)	31 (16.8)	1
Sex			0.426
Male	10 (26.3)	35 (19.0)	
Female	28 (73.7)	149 (81.0)	
ASA 3+	29 (76.3)	32 (17.4)	<0.001
Atrial fibrillation	7 (18.4)	2 (1.1)	<0.001
Asthma	3 (7.9)	14 (7.6)	1
Anxiety	8 (21.1)	28 (15.2)	0.518
Anemia	3 (7.9)	2 (1.1)	0.048
CAD	6 (15.8)	3 (1.6)	<0.001
CHF	6 (15.8)	1 (0.5)	<0.001
Coagulation defects	0 (0)	1 (0.5)	1
COPD	5 (13.2)	1 (0.5)	<0.001
Dementia	5 (13.2)	1 (0.5)	<0.001
Depressive disorder	9 (23.7)	22 (12.0)	0.101
ESRD	7 (18.4)	1 (0.5)	<0.001
GERD	13 (34.2)	27 (14.7)	0.009
HTN	18 (47.4)	47 (25.5)	0.013
Liver disease	1 (2.6)	4 (2.2)	1
Malnutrition	1 (2.6)	0 (0)	0.382
Mania/bipolar	3 (7.9)	3 (1.6)	0.106
Neoplasm	1 (2.6)	3 (1.6)	1
Osteoarthritis	5 (13.2)	27 (14.7)	1
Obesity/overweight	13 (34.2)	37 (20.1)	0.093
Rheumatoid arthritis	2 (5.3)	2 (1.1)	0.275
Sleep apnea	7 (18.4)	8 (4.3)	0.005
Type 1 diabetes	1 (2.6)	1 (0.5)	0.766
Type 2 diabetes	15 (39.5)	12 (6.5)	<0.001
Vascular disease	1 (2.6)	0 (0)	0.382
Pre HCC score	1.03 ± 1.11	0.38 ± 0.12	0.006

Those who were inpatients had their surgery on average five days sooner than outpatients (4.50 ± 6.11 vs. 9.83 ± 4.48; p < 0.001), while outpatients had a greater proportion of patients that had a preoperative delay greater than seven days (67.9% (125) vs. 18.4% (7); p < 0.001). Further, inpatients were more likely to undergo surgery in less than 48 hours (42.1% (16) vs. 2.2% (4); p < 0.001) and had a shorter median time from injury to surgery (two days vs. nine days; p < 0.001). Those who were inpatient had a higher proportion of patients who were functionally dependent (7.9% (3)) and partially dependent (42.1% (16)), while those who were outpatient had a higher proportion of patients who were functionally independent (96.7% (178); p < 0.001). Finally, there were no significant differences in the injury mechanism and the reason for preoperative delays (Table 2).

**Table 2:** Injury and surgical details Data are expressed as mean ± SD or n (%); p-values < 0.05 in bold

Injury details	Inpatient (n = 38)	Outpatient (n = 184)	p-value
Injury mechanism type			0.086
Fall	6 (15.7)	50 (27.2)	
Low energy	29 (76.3)	105 (57.1)	
High energy	3 (7.9)	29 (15.8)	
Time injury to surgery (days)	4.50 ± 6.11	9.83 ± 4.48	<0.001
Surgery before 48 hours	16 (42.1)	4 (2.2)	<0.001
Median time injury to surgery	2	9	<0.001
Preop delay (>7 days)	7 (18.4)	125 (67.9)	<0.001
Preop delay			0.184
Skin compromise	4 (57.1)	33 (26.4)	
Swelling	3 (42.9)	92 (73.6)	
Functional status			<0.001
Dependent	3 (7.9)	0 (0)	
Partially dependent	16 (42.1)	6 (3.3)	
Independent	19 (50.0)	178 (96.7)	
Minutes in the operating room	154.68 ± 20.37	140.10 ± 48.11	0.055

Postoperatively, those who were inpatient had a longer length of stay (5.21 ± 4.17 days vs. 0.08 ± 0.51 days; p < 0.001), fewer patients discharged home (36.8% (14) vs. 98.9% (182); p < 0.001), and more patients return to the ED within 90 days (18.4% (7) vs. 3.3% (6); p = 0.002) than outpatients. Those who were outpatient, on average, had a longer time to reoperation (11.48 ± 14.20 months vs. 1.50 ± 1.23 months; p < 0.001) and had a longer orthopedic follow-up (553.61 ± 662.58 months vs. 318.71 ± 402.69 months; p = 0.005). There were no significant differences between inpatients and outpatients for time to ED visit, rate of 90-day readmission, time to readmission, rate of unplanned reoperation, any reoperation, postoperative complication, complication type, or negative outcome (Table 3).

**Table 3:** Postoperative outcomes Data are expressed as mean ± SD or n (%); p-values < 0.05 in bold ED, emergency department; I&D, incision and drainage; ORIF, open reduction and internal fixation; DVT, deep vein thrombosis; PE, pulmonary embolism

Postoperative outcome	Inpatient (n = 38)	Outpatient (n = 184)	p-value
Length of stay (days)	5.21 ± 4.17	0.08 ± 0.51	<0.001
Discharge home	14 (36.8)	182 (98.9)	<0.001
90-day ED visit	7 (18.4)	6 (3.3)	0.001
Time to ED visit (days)	41.14 ± 30.99	20.00 ± 17.18	0.154
90-day readmission	3 (7.9)	5 (2.7)	0.287
Time to readmission (days)	14.33 ± 2.31	33.20 ± 35.44	0.300
Any reoperation	2 (5.3)	36 (19.6)	0.052
Unplanned reoperation	2 (5.3)	14 (7.6)	0.333
Time to reoperation (Mo.)	1.50 ± 1.23	11.48 ± 14.20	<0.001
Reoperation reason			<0.001
Hardware removal	1 (2.6)	33 (17.9)	
I&D	0 (0)	1 (0.5)	
Revision ORIF	0 (0)	2 (1.1)	
Other (femoral-tibial vein bypass)	1 (2.6)	0 (0)	
Postoperative complication	7 (18.4)	35 (19.0)	1
Complication type			0.834
Wound healing	5 (13.2)	22 (12.0)	
Non-union	1 (2.6)	3 (1.6)	
DVT/PE	0 (0)	2 (1.1)	
Other (pain, arthritis, tendinitis, tendon injury)	1 (2.6)	8 (4.3)	
Any negative outcome	11 (28.9)	56 (30.4)	1
Months to the last orthopedic follow-up	318.71 ± 402.69	553.61 ± 662.58	0.005

Multivariate logistic regression showed that neither age, BMI, ASA 3+, outpatient status, nor the time to surgery was a predictor of a negative outcome following trimalleolar ankle ORIF (Table 4).

**Table 4:** Multivariate logistic regression: predictors of negative outcomes in outpatient treatment of patients following ankle ORIF Negative outcome: 90-day ED visit or readmission, complication, or unplanned reoperation; p-values < 0.05 are in bold BMI, body mass index; ASA, American Society of Anesthesiologists score; ORIF, open reduction and internal fixation

Predictors	Odds ratio	95% confidence interval	p-value
Age	0.99	0.97 to 1.00	0.729
BMI	1.01	0.99 to 1.02	0.626
ASA 3+	1.70	1.00 to 2.88	0.177
Outpatient	1.11	1.00 to 1.24	0.825
Time to surgery	1.05	0.99 to 1.11	0.091

## Discussion

As demonstrated in our results, patients with ankle fractures managed as an inpatient were older, had higher BMIs, and had more comorbidities than those discharged and treated with outpatient surgery. Inpatient status was shown to lead to fewer surgical delays than outpatient management. Despite their higher risk profile, patients admitted from the ED and treated as inpatients did not experience increased rates of reoperations or complications. Given the potential benefits of decreased delays to surgery, we suggest a lower threshold for admitting and operating on trimalleolar fractures as inpatients may be warranted.

Multiple prior studies have evaluated the time from injury to surgery in an attempt to determine the optimal timing of surgery for trimalleolar ankle fractures. A retrospective review of 205 closed ankle fractures from January 1, 2004, to December 31, 2009, treated surgically at a single institution demonstrated significantly higher complication rates in patients experiencing a delay in treatment than those who received fixation within one day [4]. This study also reported significant differences in patient-reported outcomes, with an 11.5-point decrease in the American Orthopedic Foot and Ankle Score (AOFAS), a 10-point decrease in the Olerud-Molander Ankle Score (OMAS), and a 0.5-point decrease in the visual analog pain scale (VAS) in the delayed group [4]. Further, the delays caused by outpatient management of ankle fractures can adversely affect outcomes. A retrospective review of 196 closed ankle fractures from April 2016 to March 2017 compared by inpatient or outpatient management showed that outpatients waited an average of 9.6 days before surgery, while inpatients underwent operative fixation in an average of 2.0 days [13]. This is similar to our results demonstrating an average time from injury to surgery of 9.8 days in cases managed as outpatients. While some patients are not appropriate for immediate surgical treatment, a lower threshold for admitting ankle fracture patients may improve outcomes by reducing delays in fixation. Surgeons should use their clinical judgment and evaluate patient factors to determine whether inpatient management would be beneficial to decrease the likelihood of complications for this injury pattern.

For both our inpatient and outpatient populations, there were no significant differences in time to ED visit, rate of 90-day readmission, time to readmission, rate of unplanned reoperation, any reoperation, postoperative complication, complication type, or negative outcome. Current studies are divided on the differences in outcomes between inpatient and outpatient ankle fracture treatment. A retrospective analysis of a prospective database at a level one trauma center evaluating 476 ankle fracture patients compared inpatient to outpatient management and demonstrated that the rate of complications and unplanned revisions were significantly higher in the hospitalized group compared to those managed as an outpatient [14]. Similarly, 7383 ankle fracture patients gathered from the American College of Surgeons National Surgery Quality Improvement Program database were evaluated based on inpatient or outpatient management. Inpatients were significantly more likely to develop severe complications, such as deep wound infections and pulmonary embolism, and minor complications than their outpatient counterparts [15]. Conversely, a retrospective review of all patients undergoing ORIF for closed ankle fractures at a single institution from 2005 to 2013 was propensity-matched and compared based on the inpatient or outpatient status at the time of surgery. No significant differences in surgical morbidity, reoperations, and readmissions were detected in the outpatient group relative to the inpatient group [16]. In our results, the lack of significant differences in outcomes between groups may indicate that post-operative management of both inpatient and outpatient fixation for trimalleolar fractures is acceptable, and appropriate surgical fixation is the most important factor in the healing of the fracture and return to pre-injury status. 

The primary limitation to admitting patients for surgical treatment of ankle fractures is the increased cost associated with longer hospital length of stay. This is a valid concern, as inpatient cases required an average length of stay of over five days, compared to zero in the outpatient cohort in our results. Prior studies have estimated that each additional inpatient hospital day incurs a cost of $1,800-$2,000 [17,18]. In a study combining institutional and national data, Stull et al. concluded that the routine admission of closed lateral malleolus, bimalleolar, and trimalleolar fractures treated with ORIF resulted in over $367 million in excess facility reimbursements in the U.S. annually [7]. Clearly, outpatient management of ankle fractures reduces the direct cost of care to insurers. However, this narrow view of cost fails to account for the indirect costs of travel and missed work for both patients and their caregivers, which are associated with the increased time to surgery and delayed initiation of rehabilitation in outpatient treatment. The burden of these costs to patients and society can be significant [19-21]; therefore, future studies comparing the cost-utility of inpatient and outpatient ankle fracture care are warranted before a definitive decision regarding the most economically efficient approach can be reached. Further, outpatient management is not the only approach that can be used to mitigate the costs of ankle fracture treatment. Across various elective and non-elective orthopedic procedures, the adoption of enhanced recovery pathways has been shown to reduce hospital LOS and cost while improving treatment outcomes [22-24]. However, a paucity of studies evaluating such pathways for ankle fracture patients specifically exists. One example of an effective ankle fracture pathway has been published by Duckworth et al. [9]. By implementing a standardized approach that included inpatient admission of unstable, high energy, and/or open fractures; medically or socially unstable patients requiring surgery; and discharge with the outpatient treatment of stable, low energy, closed fractures, the authors demonstrated a cost per case reduction of 18% over three years. Given the potential benefits of inpatient treatment of trimalleolar ankle fractures, continued evaluation and refinement of treatment pathways are warranted to optimize the quality and cost of care in this population. 

There are multiple limitations to this retrospective study. By only utilizing data from a single institution and, therefore, a small sample size, our results may not be applicable to larger trauma centers or healthcare systems with a more variable population. Similarly, our patient population may not represent populations in other parts of the country; significant differences in comorbidities such as obesity or type one diabetes may exist that would yield differing results than ours. Additionally, the Lauge-Hansen and Weber classification systems were not utilized, and all ankle fractures were classified by Current Procedural Terminology (CPT) codes. This may have led to oversimplification of fracture patterns and their representation in the data. A larger study with a standardized protocol for the management of trimalleolar fractures may be better powered to detect differences in outcomes between the groups and further guide surgeons in the decision process for the management of these fracture patterns. Finally, our study did not evaluate patient satisfaction with the care experience. To our knowledge, no study has compared patient satisfaction between inpatient and outpatient management of ankle fractures to date. However, given the challenges of performing activities of daily living and managing pain at home while waiting for surgery, we hypothesize that admitted patients likely have a more favorable perception of their overall care experience. Formal evaluation of satisfaction between these populations presents an opportunity for future study.

## Conclusions

Inpatient admission of patients presenting with trimalleolar ankle fractures resulted in faster time to surgery and fewer surgical delays than outpatient surgery. Despite having more preoperative risk factors, inpatients experienced similar postoperative outcomes as patients discharged home to return for outpatient surgery. Less restrictive admission criteria may improve the patient experience by providing more patients with support and pain control in the hospital setting while decreasing the time to surgery.
